# Reasons why selected young female and male football players drop out on their path to the elite senior level

**DOI:** 10.3389/fspor.2025.1703101

**Published:** 2025-11-19

**Authors:** Peter Brusvik, Tor Söderström

**Affiliations:** Department of Education Umeå University, Umeå School of Sport Sciences, Umeå University, Umeå, Sweden

**Keywords:** drop-out behaviour, talented football players, age, gender, soccer

## Abstract

**Introduction:**

This study examines the reasons why female and male players, selected to a nationwide youth football talent programme (regional youth teams), dropped out of football between 15–23 years of age.

**Methods:**

The study builds on the answers provided by 363 women and 170 men to an open-ended question on a questionnaire: “*Can you describe in your own words why you stopped playing club football?”*

**Results and discussion:**

The analysis showed that for both women and men, structural reasons were the most common motives for dropping out, followed by interpersonal and intrapersonal reasons. Although the analysis did not show any significant differences in dropout constraints between genders, the significance of different reasons for dropping out within these constraint dimensions varies between women and men. For women, there is a gradual shift from injuries causing the end of a football career in the age group 15–19 years to a perceived lack of time and an increased focus on education and working career. Among men, with an increase in age, injuries, studies, and work become the main reasons for dropping out of football, although not as clearly as among women. Moreover, the findings emphasize that the reasons for dropping out interact with each other, and some prevent while others support continued play. Understanding the reasons why selected talented players dropped out of football can provide guidance for sustainable talent development, thereby reducing elements that influence dropout behavior among talented youth football players.

## Introduction

To reach an elite level in football, each country has regional and national initiatives to identify, select, and develop youth players [cf. (1)]. Although significant resources are invested in the football development of these young individuals, most of them do not reach the elite level. A significant proportion of these youth players, especially girls, drop out of football before they reach the senior level (2, 3). To date, the available knowledge on dropouts from organized team sports is primarily based on quantitative studies of adolescents [e.g., (4)] who participate in organized club-based sports [e.g., (5)]. However, we know significantly less about the dropout behavior among those boys and girls who are seen as particularly talented and have been selected for extracurricular talent development environments, which this study aims to explore.

Thus far, the research on the dropout behavior of children and youth in organized sports in general has revealed that dropping out of sports is primarily related to their internal state and attributes, namely, interpersonal constraints. In a football context, activity-related motivation (lack of enjoyment) [e.g., (6, 7)], perceived competence and skills [e.g., (8–11)], and internal pressure have been linked to dropout behavior [e.g., (12–14)]. Research has also highlighted that social interactions within an individual's environment—namely, interpersonal constraints, such as pressure from others in both sport and social circumstances, poor team culture, and the consequences of selection—influence dropout behavior from sports [e.g., (11, 12)]. Having other things to do outside of sports has also been noted to be a central reason for dropping out, which becomes more common with age (15), especially among girls (6). Other reasons found in the literature that influence dropping out are structural constraints, such as “time priorities” and “injuries” [e.g., (12, 16)]. Time priorities are a decisive dropout factor because training and competition take time in relation to, for example, school (11, 16, 17). The second most common structural constraint, injury, is often a career-ending dropout reason. A study on Norwegian track and field athletes found that adolescent athletes identified as talents dropped out mainly because of injuries (18).

Scholars have also revealed that the age of the athletes is a decisive aspect in dropout behavior (19). Balish et al. (20) argued that there is clear evidence that age is a factor in dropping out and that children are inclined to stop playing sports as they grow older [cf. (4, 21)]. The possible explanations for this are that age influences the perception of, for example, sporting competence and that increased age affects self-determined motivation (5, 11, 22). However, regarding the focus of this study on selected athletes, Calvo et al. (54) stated that perceived competence among youth federation players in Spain was not a significant predictor of sport continuance. Regarding gender differences in relation to dropout behavior, the number of studies on women is limited (23). The studies that focus on dropout behavior from a gender perspective reveal a higher dropout rate among girls and also among those identified as talented (2, 18), more injury-related dropping out among girls (6), and the higher significance of relationships with teammates and coaches for girls compared to boys [for more details, see (16, 24)].

Overall, the research reveals that there is no single explanation for sports dropout behavior. Instead, intrapersonal and interpersonal factors, as well as structural factors, coexist and interact, often reinforcing each other and contributing to sports dropout behavior (7) and to the perception that sport is no longer as fun and meaningful as it once was [e.g., (16, 25)].

Although this body of research provides important knowledge, especially on intrapersonal reasons (e.g., basic psychological needs) for dropping out of team sports [see reviews from Back et al. (21), Balish et al. (20)], there are questions that nevertheless need to be explored. First, most reported research on dropout behavior focuses on adolescent boys, and remarkably few studies have focused on gender comparisons [e.g., (10, 55)]. Second, there is limited knowledge on age differences in the causes of dropout and how dropout trajectories change, although researchers have called for studies that investigate how age affects reasons for dropout [e.g., (10, 55, 56)]. This lack of information surrounding both the gender and age perspectives contributes to the dearth of knowledge on dropout during crucial transitions between child and junior levels and between junior and senior levels in general as well as transitions for promising and future elite athletes. Third, the applied quantitative approach has largely overlooked the voices of athletes on factors that have influenced their decision to no longer participate in sports [cf. (11)]. Finally, the emphasis on regular organized sport participation and dropout among scholars has contributed to limited knowledge of dropout behavior among boys and girls who are identified as particularly talented and selected for extracurricular talent development environments. Being chosen to participate in a talent development program is motivating and energizing for players and provides access to a training environment run by educated leaders—a privileged position compared to those who are not selected (26).

In the present study, we therefore aim to overcome limitations in previous research and investigate why selected female and male players, despite being in a privileged position (e.g., high football competence, support structures, training environment), drop out of football.

In this study, women and men who, at the age of 15, were selected to participate in the Swedish nationwide youth talent program in football will be the object of study. The nationwide program is an extracurricular development program conducted each year by the Swedish Football Association (SvFF) through football districts. It works as the recruiting base for the national U15 teams. By drawing on retrospective written narratives from selected women (363) and men (170) on why they stopped playing club football, the aim of this study is (a) to examine the underlying factors associated with dropout behavior among youth players selected for a nationwide talent program and (b) to determine how these factors can be understood in relation to age and gender*.*

Understanding the dropout trajectories of talented and selected boy and girl football players —and the reasons for dropping out—contributes to knowledge on crucial transitions that selected young football players encounter during their path to senior elite levels. This knowledge may be important to stakeholders at the national and regional levels to inform the growth of sustainable talent development environments.

## Method and analysis

### Football districts talent development

This study is part of a larger project that investigates the development paths of selected football players. Football team activities for the 24 districts of Sweden are initiated when players are approximately13 years old. The districts use a selection process for players are 13 years old, and hundreds of boys and girls participate in the training camps in each of the 24 districts (which was amended to 23 in the year 2021). During these camps, coaches gradually select 16 girls and 16 boys they believe are most talented and have the potential to develop into elite football players at 15 years of age for the district team. It is from this pool of players in the 24 districts that they players for the youth national squads (girls and boys) are selected.

### Data collection

This study uses retrospective data from a questionnaire that was sent by post to all 8,000 players who participated in the SvFF nationwide talent development program between 2001 and 2011 (players born between 1986 and 1996). Information on these players was taken from the SvFF register system, and current addresses were obtained from the population register. Every player received a letter that provided information about the project, with a link to an online questionnaire and a personal code. The participant codes were stored separately from the questionnaire, and data were processed anonymously. After one reminder, 396 men (39.6%) and 630 women (63.1%) answered the survey (*N* = 1,026), which corresponds to a response rate of 10.0% for men and 15.5% for woman. The respondents answering the survey were between 22 and 32 years of age when the data were collected. An analysis of the response rate shows that the sample is representative of the 8,000 selected football players, deviating only in having a larger proportion of women and participants born in 1986, 1987, or 1989 (*p* < 0.05). The sample was representative of the population in terms of the number of players born in respective birth quartiles (for girls, boys, and in total, *p* > 0.05), the number of players responding from each district (for girls, boys, and in total, *p* > 0.05), and the number of girls responding from each cohort (*p* > 0.05). The data presented in this study were part of a project that encompassed additional topics beyond the score of this study [see (27)]. The web-based questionnaire collected information on personal life (e.g., education level), sociocultural background (e.g., birthplace), previous involvement in sports, experience of playing football in different settings, and reasons for starting or dropping out of football. This study uses written narratives on reasons for dropping out.

### Written narratives on reasons for dropout

Of the 1,026 players who responded to the questionnaire, 686 had dropped out of organized football, with a 74% dropout rate among women (*N* = 465) and 56% among men (*N* = 221). These players answered the following question: “Can you describe in your own words why you stopped playing club football (i.e., the process that led to ending your football career)?” The responses yielded about 55 pages of text.

### Coding and analysis of written narratives

The descriptions provided by players of the constraints that influenced their decision to stop playing club football were initially sorted into three age groups (15–19, 20–24, and +24 years). The age groups were defined in conjunction with data from the SvFF national football developers and the player education plan. Narratives from players in the age range of 15–23 years were chosen for further analysis because it is during this time that players move from youth to senior football (see Table 1). Among the 686 players who had quit playing organized football (67%), 279 players (27%) stopped playing organized football between the ages of 15 and 19 years (198 women and 81 men, corresponding to a dropout rate of 31% among women and 20.5% among men answering the survey). At the age range of 20–23 years, 254 (25%) of the players (165 women and 89 men) had dropped out (26% dropout rate among women, 22.5% among men answering the survey). The players who dropped out at the age of 24 years or older (102 women and 51 men) were not included in the study as they are established themselves in senior football (see Table 1).

**Table 1:** Age when dropout occurred among selected players.

Age group (years)	Explanation	Women		Men		Total	
		*n*	%	*n*	%	*n*	%
15–19	Junior soccer^a^, district team cup, and upper secondary soccer program	198	31	81	20.5	279	27
20–23	Establishment phase in senior soccer	165	26	89	22.5	254	25
24+	Established in senior soccer	102	16	51	13	153	15
Sum		465	74	221	56	686	67

Age groups are based on general player education plan in Swedish soccer.

a Girls' junior soccer is not available in all districts. For this reason, it is common that girls wanting to continue playing soccer must try out for a senior women's soccer team.

First of all, the open-ended responses (narratives) were analyzed using content analysis (28, 29) to identify and categorize the main reasons why selected players stopped playing football. In this step, two researchers independently identified the dimensions mentioned in each open-ended response from men who dropped out at the age range of 20–23 years (89 responses). The inductive categorization of the underlying reasons was thereafter analyzed through a leisure constraints theoretical (LCT) lens, including structural, interpersonal, and intrapersonal constraints (30, 31). The interpretations of the open-ended responses followed a principle where reasons from one individual could be related to one or more of the constraint dimensions in the LCT framework. The narratives of the players reveal that the reasons for dropping out in most cases were numerous and interrelated with each other; in other words, it was not possible to interpret one main reason leading to dropout based on these data, except for those individuals who only reported a serious injury that ended continuation.

Second, the interpretations of the answers by the two researchers were compared to ensure consensus in the coding process and interrater reliability. The inductive creation of categories related to structural reasons was consistent, while the categories related to interpersonal and intrapersonal aspects were discussed to develop a valid and reliable analysis of the responses. For example, negative feelings toward the team or coach could be seen as an intrapersonal (12) or interpersonal reason (11). In this study, these types of feelings were viewed as intrapersonal because they arise in a relationship, for example, when an individual does not get along with the coach. Intrapersonal reasons—“[…] internal states and characteristics that are accountable for the development of sporting preferences and priorities” (11, 178)—were classified as internal stress or sport-specific (e.g., perceptions of sport competence). Unlike in Jackson et al. (32) and Schlesinger et al. (11), the classification of other interests followed Crane and Temple's (12) view that dropout relates to social factors or interaction with others and therefore is an interpersonal reason. Moreover, structural reasons—“[reasons that] interfere or disrupt the connection between preferences and participation such as insufficient funds to participate” (12, 117)—were viewed from a perspective of injury or lack of time [e.g., (12)]. The work in interpreting and classifying the individual descriptions of reasons for dropout resulted in subdimensions within the structural, interpersonal, and intrapersonal dimensions (Table 2).

**Table 2:** Dropout constraints among women and men aged 15–19 and 20–23 years (%).

Constraint dimensions	Subdimensions	15–19 years		20–23 years				
		Women	Men	Women	Men	*n* ^a^	Each subdim.^b^	Total^c^
Structural	Lack of time	17	14	32	22	195	45	22
	Injury	21	17	19	23	180	41	20
	Earn a living	3	1	5	4	31	7	3
	Illness	2	5	3	3	27	6	3
	Club structure	1	1	0.4	0	5	1	1
Interpersonal	Other sports	13	18	4	1	82	27	9
	Team culture	11	5	8	9	78	26	9
	Other interests	7	9	9	8	75	25	8
	Boundaries/selection	3	9	2	5	36	12	4
	Pressure (sports)	5	3	3	1	28	9	3
Intrapersonal	Lack of enjoyment/motivation	12	11	10	12	100	63	11
	Lack of competence	2	6	3	8	35	22	4
	Pressure (intrinsic)	3	2	3	3	23	15	3

a Number of players classified in each subdimension.

b Subdimension percentage within each constraint dimension (each dimension makes up 100%).

c Subdimension percentage for all constraints.

Third, one researcher (the main author) interpreted, coded, and classified the remaining answers from the women and men who dropped out in the age range of 15–19 years and from women who dropped out in the age range of 20–23 years using the developed coding principle, which considered the cocreated subdimensions (Table 2).

Fourth, an analysis was performed to understand how players negotiated various structural, interpersonal, and intrapersonal constraints and how these affected dropout behavior in football in relation to gender and age. This analysis is not comprehensive but identifies overall patterns of reasons and provides a general description of how these reasons are interrelated and cause a diminished sense of meaningfulness, leading to the decision to stop playing club football.

### Statistical analysis

The coded narratives were statistically analyzed using IBM SPSS Statistics version 26. For comparisons of the proportions of different dimensions (structural, interpersonal, intrapersonal) between girls and boys, and the different age groups, the chi-square test was used in addition to Cramer's V measure (*w*) for effect sizes (33), following Cohen's suggestions for small (*w* = 0.10), medium (*w* = 0.30), and large effect sizes (*w* = 0.50).

### Ethical approval

The study followed ethical principles of research, and written consent was collected via a letter before participants answered the survey. The study has been approved by the regional review board (dnr: 2018/68-21).

## Findings

The analysis of the 533-player narratives shows that structural, interpersonal, and intrapersonal constraints are present among the reasons for dropping out. The analysis also shows similarities as well as differences between genders and different ages within the constraint dimensions (see Table 2).

Table 3 shows that structural reasons (49%) (e.g., injury) for dropping out were the most common, followed by interpersonal (33%) (e.g., other sports) and intrapersonal reasons (18%) (e.g., lack of enjoyment). Table 2 also shows that the subdimensions mentioned most often were lack of time (*n* = 195), injury (*n* = 180), and lack of enjoyment (*n* = 100).

**Table 3:** Drop-out constraints, women and men, aged 15–19 years and 20–23 years, frequences and proportion of all answers.

Constraints	15–19 years				20–23 years				Overall^a^					
	Women^b^		Men^c^		Women		Men		15–19 years		20–23 years		All	
	*n*	%	*n*	%	*n*	%	*n*	%	*n*	%	*n*	%	*n*	%
Structural	145	44	49	37	165	59	79	53	194	42	244	57	438	49
Interpersonal	132	39	59	44	72	26	36	24	191	41	108	25	299	33
Intrapersonal	56	17	25	19	43	15	34	23	81	17	77	18	158	18

a Significant difference between age groups 15–19 years and 20–23 years [χ^2^(2) = 27.37, *p* < .05, w = .17].

b Significant difference between women, age groups 15–19 years and 20–23 years [χ^2^(2) = 16.18, *p* < .05, w = .16].

c Significant differences between men, age groups 15–19 years and 20–23 years [χ^2^(2) = 13.11, *p* < .05, w = .22].

Table 3 shows that, although there were minor differences between genders regarding constraint dimensions, there were no significant differences in dropout dimensions between genders, overall, or by age group (*p* > 0.05).

Table 3 further shows that the reasons for dropout change over time. The chi-square analysis shows that, although with a small-to-medium effect size, there is a significant difference between the age groups 15–19 and 20–23 years, overall (*p* < 0.05, *w* = 0.17), for women (*p* < 0.05, *w* = 0.16), and for men (*p* < 0.05, *w* = 0.22). In the age group of 15–19 years, structural and interpersonal reasons are equally common, while structural reasons are more common in the narratives of older players (20–23 years).

The following sections present what the dropout constraints illustrated in Table 2 mean for women and men at different ages.

### Structural constraints in youth and young adulthood

Some important structural dropout reasons illustrated in Table 2 for both women and men at different ages are lack of time and injuries. Lack of time, while common already at the age range of 15–19 years (women 17% and men 14%), is mentioned more often as a reason for dropout at the age range of 20–23 years and especially among women (women 32% and men 22%). As noted in other studies (11, 16, 17), this constraint relates to football interfering with other activities the players wanted to pursue, as well as a lack of time to train and play football due to studies and/or work: “[I] started working shifts and therefore missed training” (Woman, 4,189, 15–19 years). As one man put forth, “It took too much time from other things I wanted to do. I wanted to study and could not see myself only playing football in the future” (Man, 4,279, 15–19 years). Overall, the responses mentioning a lack of time reflect the fact that pursuing elite football is challenging if one also wants to pursue studies and make a living upon entering adulthood.

Injury as a reason, identified in previous studies as crucial [e.g., (11, 12, 34)], could relate to either an injury that ended the career immediately—“[I] had to undergo a total of three cruciate ligament surgeries, but my knee never got good enough to return to football” (Woman, 4,171, 20–23 years)—or an injury that occurred as a consequence of the physical load over time. One woman writes that she had a serious foot injury caused by playing on both the girls’ and senior teams, which required her to participate in more practices. She underscores the following:

[…] to the doctors, this injury probably occurred when there was so much pressure on us girls when we went up in the current Division 2 at the age of thirteen and then played for×number of teams at the same time and juggled ten, eleven practices plus matches each week (Woman, 4,011, 15–19 years).

The injury rate in this study appears to be higher and a more significant reason for dropping out than what previous studies noted [e.g., (4, 12), Schlesinger et al. (11)]. It is most likely a consequence of the fact that being selected as a talent and included in the district squad at the age of 15 years leads to more training and matches with different teams [for more details, see (18)]. A possible explanation for the higher injury rate in women in the age range of 15–19 years is that there are fewer junior teams for girls in Sweden and consequently early establishment of players at the senior level. The higher injury rate among men aged 20–23 years suggests that to the challenges faced by women aged 15–19 years may occur to a greater extent among men aged 20–23 years when they are establishing themselves at the senior level.

The structural constraints that have lesser influence on the decision to drop out are illness (3%) (mental, e.g., eating disorder/depression, or physical, e.g., migraines) and the need to earn a living (3%) [for more details, see (35)]. Although earning a living is not a frequently reported reason in this study (see Table 2), a gender difference is seen. Young women (e.g., 15–16 years) often realize, as one woman points out, that “you cannot envision a future as a professional women's football player […]” (Woman, 4,629, 20–23 years) and that it will be difficult, even if they reach an elite level, to “support yourself in football” (Woman, 4,308, 15–19 years). The responses of men in this subdimension are more related to playing at a level too low to “make money on football” (Man, 4,912, 20–23 years), which becomes clear when they are older (20–23 years). In most cases, earning a living interacted with other reasons that ultimately resulted in dropout.

### Interpersonal constraints in youth and young adulthood

Table 2 shows that interpersonal reasons are the second most common constraint dimension for players in this population (33%). Reasons related to choosing other sports, other interests, and team culture are the most common in the narratives. Overall, these account for approximately 8%–9% of the reasons why 533 players dropped out. Choosing other sports is the most common dropout reason for men (18%) and the third most common reason for women (13%) aged 15–19 years.

The choice of other sports may be based on a feeling of greater competence in another sport, which has been noted in previous research [e.g., (8, 57)]: “[…] the choice fell on floorball as I felt I was better at it than football” (Woman, 4,016, 15–19 years). Choosing another sport can also be related to not gaining confidence, getting little playing time, or aspects related to coaching decisions:

[…] I played a yy match instead of attending a practice with the xx (football) team. After that, I was no longer welcomed to the xx team because I played a yy match when it was yy season. This made me really fed up with football and led me to choose yy as a specialization in upper secondary school instead of football (Man, 4,305, 15–19 years). (The placeholders yy and xx are for confidentiality reasons).

Interest in other activities as a reason for dropping out has roughly equal importance among women and men at the age ranges of 15–19 and 20–23 years (8%). In that sense, the results do not support previous research suggesting that there is a more significant reason why women drop out (6) or that it becomes more common the older an individual gets (15). The narratives indicate that other interests reflect situations when other things are perceived as more fun than football. One man emphasizes that “Dance and music became an increasingly important factor in my life, while I became more and more unmotivated for football” (Man, 4,012, 15–19 years). Both younger and older players found other things more fun or “exciting and interesting” (Woman, 4,056, 15–19 years) to do than football, which became a decisive dropout reason: “I didn't think football was as fun anymore and I didn't think it was worth the time it took. I'd rather do other things in my spare time” (Woman, 4,267, 20–23 years).

The third most common interpersonal reason is team culture (9%), which previous research acknowledges in terms of, for example, poor relationships with teammates or coaches (12, 17). Team culture is a somewhat more common reason for dropping out among women (11%) than men aged 15–19 years (5%) (11, 16), but equally common at the age range of 20–23 years. However, team culture for this cohort of players is not primarily about poor relationships and negative coaching experiences in a team, as highlighted by other research, but a consequence of their football skills, resulting in them being moved between several teams and not feeling at home anywhere.

According to Calvo et al. (54), the narratives express a lack of connection with the team, which influences dropout decisions: “[…] jumping between the different teams made it difficult for me to become socially involved in each of them” (Woman, 4,105, 15–19 years). Similarly, as a man points out, “No really clear team affiliation made football more boring. Jumped back and forth between U21 team and training with the A-team from about 15–16 years of age” (Man, 4,353, 15–19 years). One woman who joined the women's team states that she was much younger than the other players and therefore had “[…] a hard time finding people that felt natural and that I enjoyed being with. I did not feel that anyone really took responsibility for me during that time […]” (Woman, 5,114, 15–19 years).

Other significant factors associated to team culture relate to how junior and senior teams interact with a perceived lack of competence among coaches, and a sense of plateauing in development:

[…] Felt a clear progress in the junior team […] it felt rewarding, and I developed. Football-wise, but also socially developing. The move to A team football did not go well as the coach was not knowledgeable and did not have the ability to contribute to my own development or to the team developing as a group (Man, 4,798, 20–23 years).

The interpersonal reasons that affect the choice of dropping out to a lesser extent are boundaries/selections (4%) and pressure (3%) (Table 2). The reason of boundaries/selections is more common among men than women, especially at the age range of 15–19 years (9% vs. 3%) and relates to the subjective values of coaches and sports groups selecting players for a team, namely, when individuals are not promoted or selected for teams and squads [for more details, see (36)]. As one man points out, “Coaches who did not want to give younger players the chance meant that you were not allowed to show your skills in the first team properly” (Man, 4,363, age 15–19).

The dropout reason related to the stress associated with extrinsic pressures from the team, coaches, or others—which previous research highlighted (11, 12, 37)—is not a common reason in this study, regardless of age or gender (3%), but is somewhat more common among women.

### Intrapersonal constraints

Table 2 shows that intrapersonal constraints (18%) are the least important constraint dimension for this cohort of players. However, lack of enjoyment/motivation (11%) is the most common intrapersonal reason to drop out for both women (12% and 10%) and men (11% and 12%), and the third most common subdimension among all players in this study. It was noted as a dominant reason for dropout even in previous research [e.g., (12, 37)]; however, few studies have described what the individual means by lack of enjoyment. The present study shows that lack of enjoyment can be either a single reason for dropping out—e.g., “I simply got tired” (Man, 4,199, 15–19 years)—or, as most commonly described, a factor that interacts with other factors (e.g., boundaries and team culture), all contributing to football no longer being meaningful:

[…] I put a lot of time and energy into football and felt that I did not get as much back in the form of playing time as I, in my opinion, should have received. This in combination led to me not thinking it was as fun anymore and therefore stopped playing […](Woman, 4,339, 15–19 years).

Lack of enjoyment can also be a consequence of the manner in which the players play the sport; in this study, players reported often playing with older teams as well as in district and upper secondary football programs or national teams. As one women pointed out, “It wasn't fun anymore. It was too much” (Woman, 4,144, 15–19 years), or, as one man highlighted, “I trained almost every day and played matches with two teams in the club so the whole weekend was matches. Matches were the fun part for the most part but training wasn't fun for me anymore” (Man, 4,031, 15–19 years). In addition, losing enjoyment may also be related to the transition from junior to senior level where previous success with more playing time and high self-confidence could have changed quickly. One man who had less playing time wrote that his motivation “[…] was destroyed in a way, there were probably more reasons, one is quite clearly that I wasn't able to handle a setback with less playing time when you had previously always played continuously” (Man, 4,538, 20–23 years).

Lack of competence (4%)—an intrapersonal reason—is not an important reason among women regardless of age (15–19 years, 2%; 20–23 years, 3%), although findings from other research have labeled it as a major reason for dropping out of football (or sports) (9, 14, 24, 38). It is somewhat more common in both younger (6%) and older men (8%). However, for men in the present study, dropout was not attributed to low perceived football competence because they had been identified as talents within a district at the age of 15. However, as one man stated, “I realized that my own football ability was far from what was required to play at an elite level” (Man, 4,420, 20–23 years); this realization affected the joy and motivation to continue playing [for more details, see (39, 40)].

Intrinsic pressure is a reason for dropout according to some reviews [e.g., (11, 12)] but is not a common subdimension (3%) in this study, regardless of age or gender (women 3% 15–19 years, 3% 20–23 years, men 2% 15–19 years, 3% 20–23 years).

## Discussion

This study adds to the literature on dropout behavior in sports by describing how structural constraints significantly influence the decision to quit among selected female and male players. The findings show that, although there are no significant differences between the selected male and female players in terms of structural, interpersonal, or intrapersonal constraints, gender-related differences exist within subdimensions and those change over time. For women, there is a gradual shift from injuries causing the end of a football career at the age group of 15–19 years to a perceived lack of time and an increased focus on education and professional career—as well as a seeming lack of opportunity earning a living from the sport—at the age group of 20–23 years [for more details, see (6, 15)]. Among men, with age, they become more interested in studies and work rather than football, although this is not as significant as it is among women. This result not only underscores how age plays into the individual's decision to quit playing football—as noted by other research (4, 20, 21)—but also offers insights into how the reasons behind leaving football vary with age.

The emphasis on selected players reveals that their dropout trajectories have a different pattern compared to the dropout trajectories noted in studies performed on regular organized sport. For selected players, the structural constraints for both women and men collectively become increasingly influential in the decision to quit or not as they get older, compared with interpersonal constraints, while intrapersonal constraints do not change over time [for more details, see (41)]. In a sense, the findings reflect what has been noted in studies on dual-career students aged 16–19 years regarding the challenges they face in terms of balancing sport, education, and personal life, which may lead some to end their athletic participation (42).

Structural constraints have a high impact on dropping out compared with interpersonal and intrapersonal constraints, as were found to be more common reasons for dropping out in earlier research [e.g., (11, 12, 20–22)]. This is most likely a consequence of the explorative study approach, the cohort of players investigated, and the fact that this study captures dropout behavior in young adulthood. The open-ended question used in the study has made it possible to capture a wider range of constraints and interrelations that are not limited to specific constraints [see Battaglia et al. (24) for the focus on intra- and interpersonal influences]. The cohort of players investigated also possess football skills that help them explain why certain dropout factors—as identified in previous research—do not apply to them. Instead, as the present study reveals, structural reasons appear to play a decisive role in why people choose to quit football. In addition, the structural constraints that dominated in this study may also be a result of previously more limited samples in terms of gender, age, number of players, and the contexts that players represent [e.g., (5, 18, 43)]. Previous studies have mainly investigated dropout behavior in youth years (20, 22). The present study demonstrates that structural reasons, such as lack of time, particularly for women, increase significantly with increasing age.

The findings of this study reveal that being recognized by others as talented results in increased training load in a more physically demanding setting during the transition from junior to senior football, namely, training and playing with both youth and senior teams. One possible explanation for the significance of the lack of time in relation to dropout behavior in this study is that this increased load becomes a normalized aspect of their football participation. The older the players get, the harder it becomes to maintain that type of training, in particular if they also want to, for example, get an education. This increased load, as the findings indicate, may also impact how much enjoyment they find in football, which influences their decision to continue playing. Moreover, as other scholars have argued (6, 44, 45), this increased training load may lead to the risk injury. Although other studies recount injuries as one reason among many for dropping out [e.g., (6, 11, 12)], particularly among selected athletes (18), the findings show that it is the main reason for dropping out for both women and men, contrary to previous research. However, further studies may provide more reliable data on whether selected players have a higher risk of injury [cf. Enoksen (18), study on selected track and field athletes], thus leading to injury becoming a greater reason to drop out compared to what previous research has revealed [e.g., (11, 12)].

The findings of this study further show that the subdimension “other sport”—at the age range of 15–19—is the most common reason among men and the third most important reason for women to drop out. This is most likely based on their admission to upper secondary school sport programs, which takes place once the players turn 16 years of age. The narratives show that these selected players often have early success across several sports; however, the selection mechanisms for elite sports force them to choose one sport (36). Typically, this choice is linked to other constraints, such as injuries and/or self-assessment of skills in relation to their perceived ability to be a starting player on a senior football team. In addition, this early success in sport also explains why the intrapersonal reason of perceived competence in sport is not considered an important reason for dropping out, especially among women, even though other studies have shown that it is an increasingly important dropout reason with increasing age [e.g., (13, 24)]. The investigated players had high football-specific competence at the age of 15, and therefore the aspect of the lack of competence is—similar to the findings of Calvo et al. (54) on Spanish male federation youth footballers—not as dominant in this study as noted in previous research.

Similarly, the findings of this study reveal that the high football-specific competence of the players also affects their relatedness to a team. For women in the age range of 15–19 years, training and playing with several teams affected how they perceived team culture, namely, their sense of belonging, which influenced their dropout decisions. Men in the age range of 15–19 years, however, more often than women, highlighted the club's way of handling selection and boundaries—namely, not being “given the opportunity” by the coaches between junior and senior football—as a significant reason for dropping out. For men, but not women, as the findings reveal, a sense of lacking competence to play at an elite level begins to arise and increases at the age range of 20–23 years, which then influences dropout decisions.

## Conclusion

This study addressed a specific gap in the literature—regarding reasons why players selected to a nationwide talent development program drop out from sports—by utilizing a large sample of talented players combined with an open-ended question. The findings reveal that the dropout behavior of selected players does not follow the same pattern as that noted in previous research on dropout behavior in regular sport. Furthermore, the qualitative approach in this study underscores, as previous qualitative studies have noted (46, 47), the fact that dropping out of sports is a complex process as the reasons interact with each other and can either counteract or exacerbate each other. For instance, lack of enjoyment typically does not occur suddenly; it arises due to a combination of factors. For example, playing in several teams affects, as the findings have shown, the sense of relatedness that gradually diminishes the sense of fun, as noted in studies such as that by Battaglia et al. (24) on withdrawal patterns and Beni et al. (48) on meaningful sport experiences.

Overall, the findings reveal that the reasons behind player dropout require further attention from the Swedish Football Association in the future. Substantial resources have been invested in these players, and the findings suggest that there are factors that can be influenced to prevent them from quitting football. The injury rate among these players is high, and general guidelines for talent development and within coach education—regarding the workload and number of training sessions and matches for players—need to be developed to reduce the risk of injury to talented players, or feelings of not belonging, causing them to quit football. Although coaches and their manner of coaching were not found to be a particular reason for dropping out, as other studies have shown [e.g., (4, 14, 17, 43)], coaches in general and coach education in particular need to take note of the situation the selected players are in, for example, playing in several teams. This most likely applies to more than just the Swedish football system, as the findings can presumably have implications for talent development and coaching of talented individuals everywhere. Highlighting the situation of talented players can lead to greater focus on their development, rather than just focusing on winning at all costs, which is what playing with older or senior teams is most likely about. Finally, it is possible to ask what the consequences of the ongoing professionalization process in women's football [cf. (49)] will be for selected women in terms of dropout behavior in the future. Will it be, as Brandt-Hansen and Ottesen (35) noted, that elite-level women football players need to establish a non-football-related career and secure an income [see also (50)], which influences the time-related priorities of young, talented women as well as their decision to quit playing? Or will it be that elite sport efforts can be reconciled with the demand for making a living upon entering adulthood? A recent study by Harrison et al. (51) of elite female soccer players supports, to a greater extent, the findings of Brandt-Hansen and Ottesen (35), as the majority of the players in the former study planned to carry out a dual career at the higher education level. However, future studies will show what the ongoing professionalization means for retaining young, talented women in football.

This is one of the few studies to date addressing the dropout behavior among selected football players. Understanding the reasons for dropping out of football as expressed by talented and selected players can provide guidance for a sustainable talent development methodology that can reduce elements that influence dropout among talented youth players in the future. A limitation of the study is the retrospective nature that may be subject to recall biases (52). However, according to Bridge and Toms (53), why a young athlete quits playing football—even though they identified as talented and enjoyed special privileges—is most likely due to a life event that is significant to the individual and therefore has a higher recall reliability. However, in future studies, a longitudinal approach will most likely provide a greater accuracy in terms of capturing the underlying reasons for dropping out from childhood to young adulthood.

Finally, this study emphasizes the importance of considering both age and gender aspects, so that efforts made to retain athletes for as long as possible are not focused solely on a specific age range or only on men.

## Data Availability

Inquiries about the data included in the study can be directed to the corresponding author, peter.brusvik@umu.se.
