# Correction: Reasons why selected young female and male football players drop out on their path to the elite senior level

**DOI:** 10.3389/fspor.2025.1750727

**Published:** 2026-01-02

**Authors:** Peter Brusvik, Tor Söderström

**Affiliations:** Department of Education Umeå University, Umeå School of Sport Sciences, Umeå University, Umeå, Sweden

**Keywords:** drop-out behaviour, talanted football players, age, gender, soccer

There was a mistake in **Table 1** as published. The “%” and “Total” columns, as well as the “Sum” row, were missing. Additionally, the caption was wrongly included as “Age groups based on the general player education plan in Swedish soccer.” The corrected table appears below.

**Table 1:** Age when dropout occurred among selected players.

Age group (years)	Explanation	Women		Men		Total	
		*n*	%	*n*	%	*n*	%
15–19	Junior soccer^a^, district team cup, and upper secondary soccer program	198	31	81	20.5	279	27
20–23	Establishment phase in senior soccer	165	26	89	22.5	254	25
24+	Established in senior soccer	102	16	51	13	153	15
Sum		465	74	221	56	686	67

Age groups are based on general player education plan in Swedish soccer.

a Girls' junior soccer is not available in all districts. For this reason, it is common that girls wanting to continue playing soccer must try out for a senior women's soccer team.

There was a mistake in **Table 3** as published. The “n” and “Overall” columns were missing. Additionally, “frequencies” was missing from the caption, and the footnotes were missing the full details. The corrected table appears below.

**Table 3:** Drop-out constraints, women and men, aged 15–19 years and 20–23 years, frequences and proportion of all answers.

Constraints	15–19 years				20–23 years				Overall^a^					
	Women^b^		Men^c^		Women		Men		15–19 years		20–23 years		All	
	*n*	%	*n*	%	*n*	%	*n*	%	*n*	%	*n*	%	*n*	%
Structural	145	44	49	37	165	59	79	53	194	42	244	57	438	49
Interpersonal	132	39	59	44	72	26	36	24	191	41	108	25	299	33
Intrapersonal	56	17	25	19	43	15	34	23	81	17	77	18	158	18

a Significant difference between age groups 15–19 years and 20–23 years [χ^2^(2) = 27.37, *p* < .05, w = .17].

b Significant difference between women, age groups 15–19 years and 20–23 years [χ^2^(2) = 16.18, *p* < .05, w = .16].

c Significant differences between men, age groups 15–19 years and 20–23 years [χ^2^(2) = 13.11, *p* < .05, w = .22].

The original version of this article has been updated.

